# Development of a predictive miRNA signature for breast cancer risk among high-risk women

**DOI:** 10.18632/oncotarget.22750

**Published:** 2017-11-28

**Authors:** Nicholas H. Farina, Jon E. Ramsey, Melissa E. Cuke, Thomas P. Ahern, David J. Shirley, Janet L. Stein, Gary S. Stein, Jane B. Lian, Marie E. Wood

**Affiliations:** ^1^ University of Vermont Cancer Center, The Robert Larner MD College of Medicine, University of Vermont, Burlington, VT, USA; ^2^ Department of Biochemistry, The Robert Larner MD College of Medicine, University of Vermont, Burlington, VT, USA; ^3^ Division of Hematology and Oncology, The Robert Larner MD College of Medicine, University of Vermont, Burlington, VT, USA; ^4^ Department of Surgery, The Robert Larner MD College of Medicine, University of Vermont, Burlington, VT, USA; ^5^ Department of Microbiology and Molecular Genetics, The Robert Larner MD College of Medicine, University of Vermont, Burlington, VT, USA

**Keywords:** microRNA, high risk breast cancer, liquid biopsy, risk signature, benign breast disease

## Abstract

Significant limitations exist in our ability to predict breast cancer risk at the individual level. Circulating microRNAs (C-miRNAs) have emerged as measurable biomarkers (liquid biopsies) for cancer detection. We evaluated the ability of C-miRNAs to identify women most likely to develop breast cancer by profiling miRNA from serum obtained long before diagnosis. 24 breast cancer cases and controls (matched for risk and age) were identified from women enrolled in the High-Risk Breast Program at the UVM Cancer Center. Isolated RNA from serum was profiled for over 2500 human miRNAs. The miRNA expression data were input into a stepwise linear regression model to discover a multivariable miRNA signature that predicts long-term risk of breast cancer. 25 candidate miRNAs were identified that individually classified cases and controls based on statistical methodologies. A refined 6-miRNA risk-signature was discovered following regression modeling that distinguishes cases and controls (AUC0.896, CI 0.804-0.988) in this cohort. A functional relationship between miRNAs that cluster together when cases are contrasted against controls was suggested and confirmed by pathway analyses. The discovered 6 miRNA risk-signature can discriminate high-risk women who ultimately develop breast cancer from those who remain cancer-free, improving current risk assessment models. Future studies will focus on functional analysis of the miRNAs in this signature and testing in larger cohorts. We propose that the combined signature is highly significant for predicting cancer risk, and worthy of further screening in larger, independent clinical cohorts.

## INTRODUCTION

Several models are available for estimation of breast cancer risk; the most widely used include the Gail [1], Claus [2], and International Breast Cancer Intervention Study (IBIS) [3] models. The Gail model uses clinical factors, biopsy history and limited family history and has been validated [1, 4]. The Claus model uses only family history [5], while the IBIS model incorporates a greater number of clinical factors and a more detailed family history [5, 6]. Each model has significant limitations and has not been found to be informative at the individual level [6]. Clearly, more precise predictors of risk are needed.

MicroRNAs (miRNAs) have emerged as promising biomarkers as they are stable in circulation and found in most body fluids [7, 8]. Circulating miRNAs (C-miRNAs) are released from almost all cells in many forms: in microvesicles [9], exosomes [10], bound to protein or lipid particles [11, 12], or unbound [13]. As such, C-miRNAs act as intercellular signaling molecules [9] and may function to establish local environments for initiation and progression of cancer. A single miRNA can simultaneously target hundreds of genes, acting as a master regulator of biological signaling pathways with established roles in controlling normal development and tissue homeostasis [14]. MicroRNAs have also been shown to have important regulatory functions on processes impacting proliferation, differentiation, and apoptosis—all of which are important for cancer development and progression [15, 16]. Thus, miRNAs may be an important and more precise biomarker of breast cancer risk.

Significant differences in miRNAs have been found between cancer patients and healthy controls [17–20], suggesting potential clinical utility for cancer detection or monitoring of disease activity [21–24]. In breast cancer patients, serum miRNA levels correlate with expression in primary breast tumors [22, 23]. It is hypothesized that C-miRNAs dysregulated in cancer patients arise from tumor tissue, although the cells of origin are unknown and differential levels may be due to host cells attempting to inhibit tumor growth. Nonetheless, C-miRNAs may provide accessible and quantitative indicators of regulatory mechanisms that predispose individuals to cancer and may be surrogates for cancer risk assessment.

We postulate that there are measurable differences in C-miRNAs within serum obtained from women at high risk for breast cancer who eventually develop tumors, and those who are at high risk, but remain cancer-free. To assess this, we analyzed global miRNA levels from serum obtained from women at high risk for breast cancer years prior to cancer development. Here we present our patient classification algorithm and a preliminary risk signature of 6 miRNAs that, when combined, discriminate cases from controls with high accuracy and precision. Although studies have provided short term risk assessment (i.e. early detection) no studies have yet evaluated the potential of liquid biopsy to predict breast cancer development years before cancer identification [25–28].

## RESULTS

### Clinical characteristics

**Figure 1:**
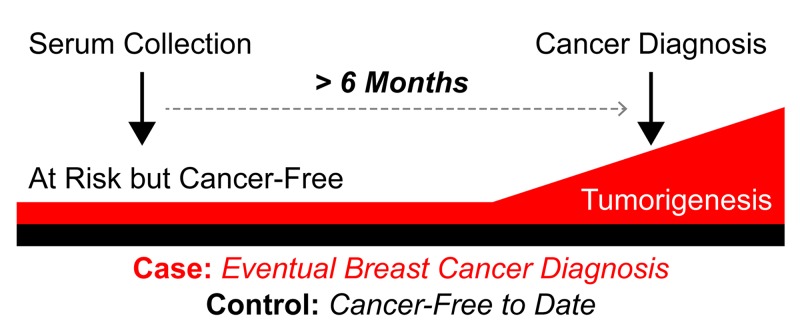
Schematic of study design and serum collection timeframe Forty-eight patients were selected from a database of 605 women at increased risk for developing breast cancer based on clinical factors: 24 women developed cancer at least six months after blood was drawn (cases) and 24 age and risk matched women who remain free of breast cancer (controls).

Twenty-four of the HRBP participants who were diagnosed with breast cancer met criteria to be included as cases. Most (66.7%) were at increased risk due to strong family history. The majority developed breast cancers that were less than 2 cm (87.5%) and lymph node negative (86.4%). Table 1 outlines the characteristics of breast cancers in this study cohort. Among cases, the median time between serum collection and breast cancer diagnosis was 3.2 years (range 0.6-8.7 years: Figure 1). Nearly 80% (19/24) of these women were diagnosed more than 15 months after serum collection. The 24 controls have been followed for a median of 10.3 years (range 4.0 -13.2 years) since serum collection, and remain cancer-free.

**Table 1:** Subject characteristics

			Affected cases (N=24)	Cancer-free controls (N=24)
Median age at blood draw* (range)			55.4 (33.9-77.5)	55.1 (32.8-78.4)
Risk factor* n (%)				
	Benign breast disease		8 (33.3)	8 (33.3)
	Family history		16 (66.7)	16 (66.7)
**Median modeled lifetime risk score** (range)				
	Gail model (n= 40)		18.4 (8.3-34.3)	19.4 (8.7-52.9)
	Claus model (n=39)		14.1 (5.0-35.8)	10.7 (5.5-27.7)
	IBIS model (n=48)		24.2 (8.1-59.6)	22.0 (10.4-49.7)
**Mammographic density at blood draw** n (%)				
	< 25%	(entirely fatty)	4 (16.7)	1 (4.2)
	25-50%	(scattered fibroglandular)	9 (37.5)	13 (54.2)
	51-75%	(heterogeneously dense)	11 (45.8)	9 (37.5)
	> 75%	(extremely dense)	0 (0)	1 (4.2)
**Ethnicity** n (%)				
	White		24 (100)	24 (100)
**Median BMI** (range)			25.2 (19.1-55.6)	25.0 (20.0-40.2)
**Charlson comorbidity index** n (%)				
	0		14 (58.3)	19 (79.2)
	1		9 (37.5)	4 (16.7)
	2-3		1 (4.17)	1 (4.17)
**Median age at diagnosis** (range)			56.9 (35.8-79.5)	
n (%)	≤ 50		7 (29.2)	
	51-59		7 (29.2)	
	> 60		10 (41.7)	
**Median no. years cancer diagnosed after blood draw** (range)			3.2 (0.6-8.7)	
**Tumor size** n (%)				
	T1 (≤ 2 cm)		21 (87.5)	
		T1a (≤ 0.5 cm)	3 (12.5)	
		T1b (> 0.5, ≤ 1 cm)	9 (37.5)	
		T1c (> 1, ≤ 2 cm)	9 (37.5)	
	T2 (> 2 cm, ≤ 5 cm)		2 (8.3)	
	T3 (> 5 cm)		1 (4.2)	
**Lymph node stage (surgical)** n (%)				
	N0		21 (87.5)	
	N1**		2 (8.3)	
	N2		1 (4.2)	
**Differentiation** n (%)				
	Well		5 (20.8)	
	Moderate		14 (58.3)	
	Poor		5 (20.8)	
**Subtype** n (%)				
	HER2- and ER+ or PR+		19 (79.2)	
	HER2+		4 (16.7)	
	Triple negative		1 (4.2)	

*Matching factor. N0: No cancer in axillary lymph nodes. N1: Cancer spread to 1-3 axillary nodes. N2: Cancer spread to 4-9 axillary nodes. Tumors classified as hormone-receptor negative if ≤ 10% cells were positive. **All areas of cancer found in these subjects’ lymph nodes were micrometastases.

### MicroRNA analysis

**Figure 2:**
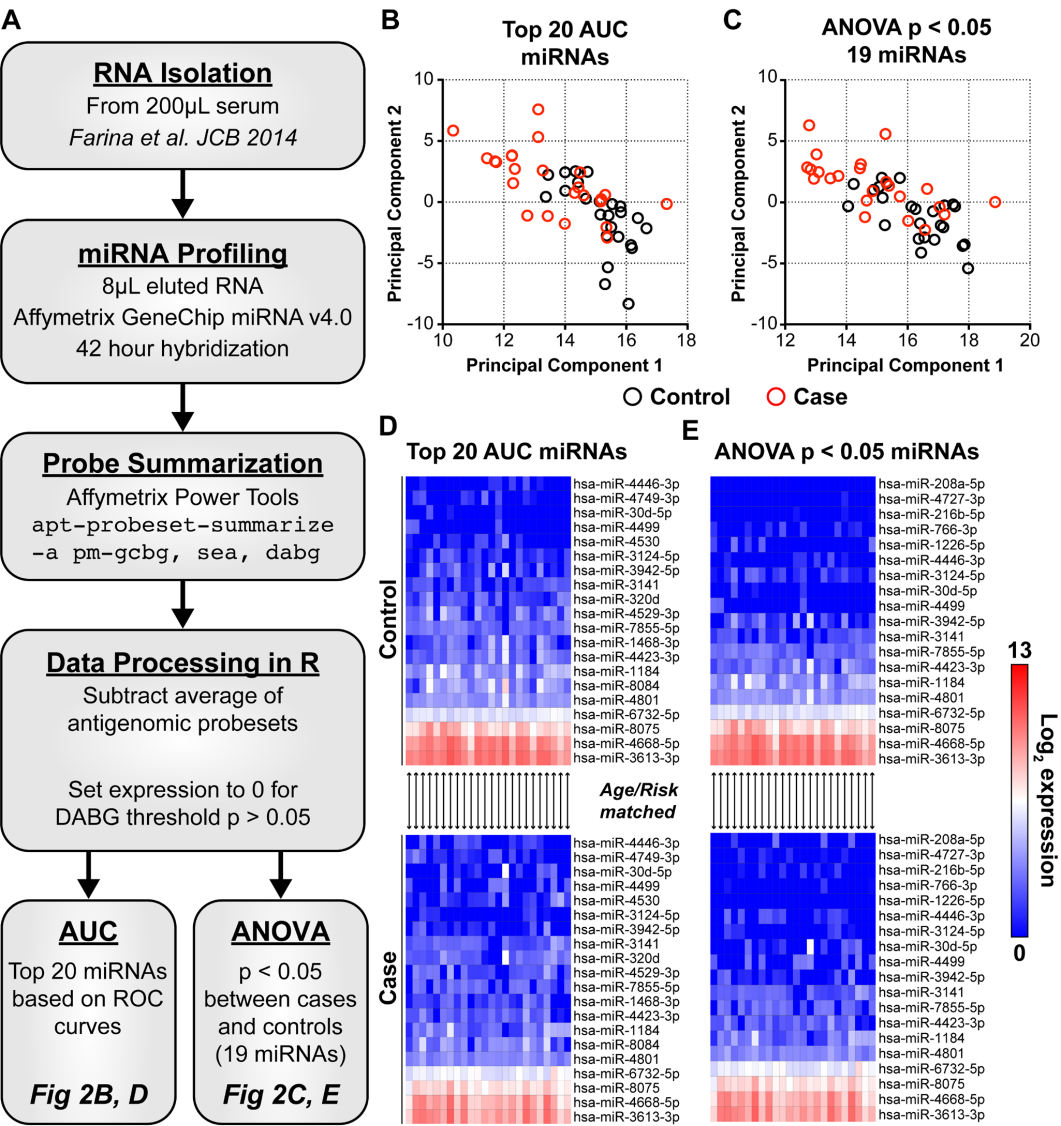
The expression of 25 candidate C-miRNAs separate women who develop cancer from those who remain cancer free Expression of all 2578 mature miRNAs within miRBase v20 were assayed in circulation. **A**: A standardized workflow was developed and optimized to reduce sample-to-sample variability that includes precise miRNA isolation, profiling, and normalization (see methods). We identified 2 sets of candidate miRNAs totaling 25 in combination. AUC: 20 miRNAs with the highest individual AUC (range 0.632-0.766). ANOVA: 19 miRNAs significantly different between cases and controls with an ANOVA p < 0.05. **B**, **C**: Principal component analysis was performed using normalized expression levels of those miRNAs within each set; top 20 AUC miRNAs or 19 miRNAs with and ANOVA p < 0.05 between cases and controls. In both instances, women with an eventual breast cancer diagnosis cluster together in the top left quadrant of the PCA graphs. Black circles represent cancer-free controls. Red circles represent eventual breast cancer cases. **D**, **E**: Heat maps show log_2_ expression of candidate miRNAs in cancer-free controls and future breast cancer cases. MicroRNAs are ordered based on hierarchical clustering (UPGMA, Euclidian distance). Each column within the stacked heat maps are matched case/control. Blue is background (0) and red is high miRNA log_2_ expression.

The levels of 2578 mature human miRNAs (miRBase v20) were interrogated in banked serum samples from the study cohort of 48 women. We developed a standardized method for serum miRNA expression analysis encompassing all steps from RNA isolation through generation of background normalized data (Figure 2A). As confirmation of this method, two different serum aliquots collected at the same time from the same woman were processed independently by different individuals and a 1:1 correlation obtained post normalization as opposed to disparate raw data (Supplementary Figure 1).

Candidate miRNAs were selected for further development of a risk signature using two distinct techniques. Area under the ROC curves (AUC), a measure of the classification accuracy, and associated 95% confidence intervals were generated for each of the 2578 interrogated miRNAs (Supplementary Table 1). Twenty miRNAs with the highest individual AUC, ranging from 0.632 to 0.766, were selected (Figure 2B, 2D). An analysis of variance (ANOVA) p-value was also calculated for each miRNA between cases and controls (Supplementary Table 2). Nineteen miRNAs were identified with an ANOVA p-value < 0.05 (Figure 2C, 2E). Combined, 25 candidate miRNAs comprise the AUC and ANOVA sets with 14 in common, 6 unique to the AUC set, and 5 unique to the ANOVA set. Principal component analysis using the expression of these 2 miRNA sets shows segregation of cases from controls (compare red and black circles in Figure 2B, 2C). In general, cases cluster to the upper left quadrant while controls trend to the lower right. Ten cases that are clearly separated in the top left of Figure 2B are the same in the top left of Figure 2C. Note that the levels of many candidate miRNAs are reduced in serum of women that ultimately developed breast cancer (compare top control heat maps to bottom case heat maps in Figure 2D, 2E), suggesting a role for these miRNAs in breast cancer risk.

### Model development

**Figure 3:**
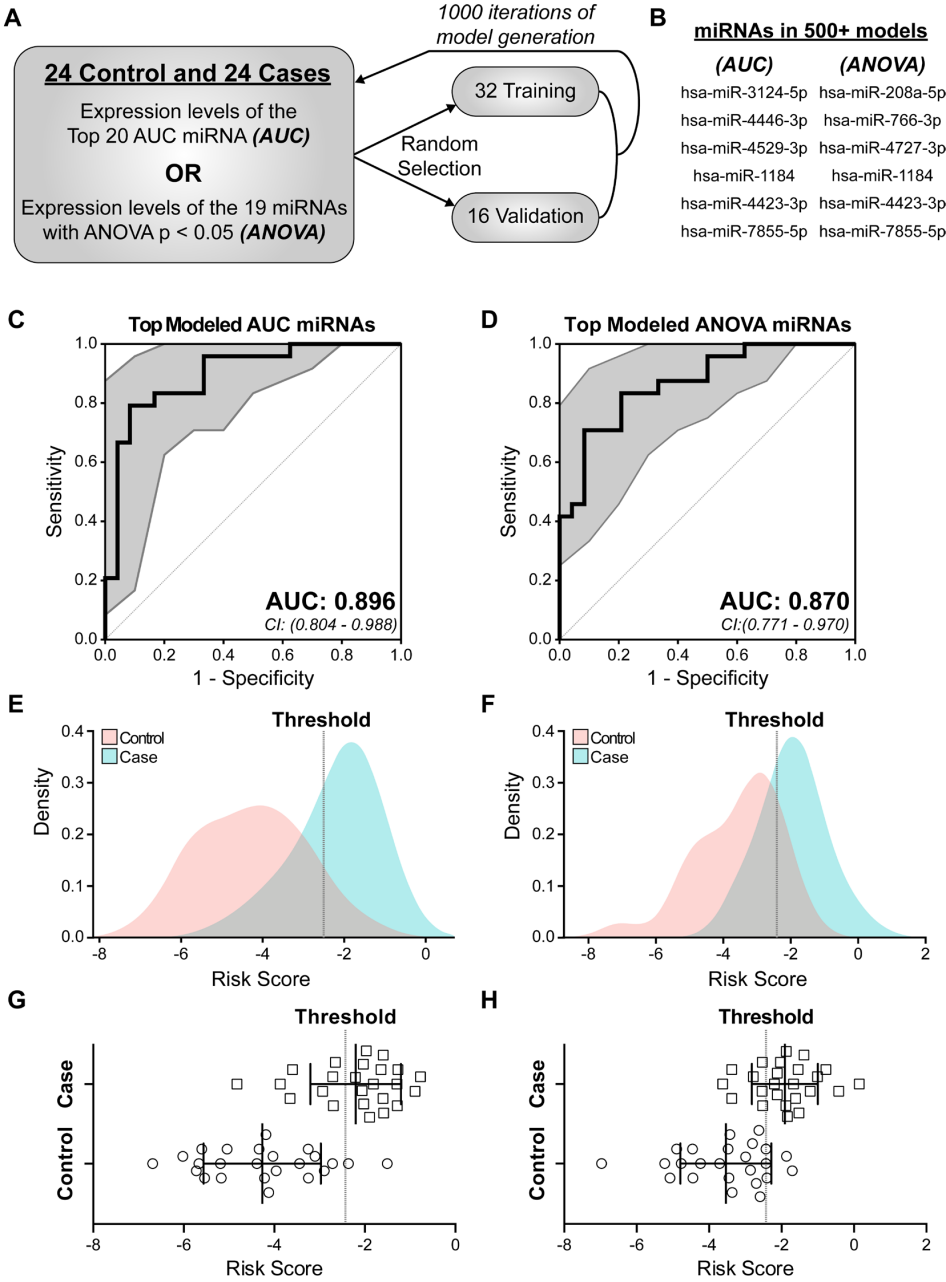
Identification of miRNAs predictive of future breast cancer diagnosis In order to develop a miRNA risk signature and score we generated a multivariable proportional hazards model based on candidate miRNA expression. Both AUC and ANOVA sets were examined. **A**: Schematic of iterative model generation using randomly selected training sets of 32 samples and corresponding validation sets of 16 samples. **B**: A total of 9 combined miRNAs were identified as signature miRNA (AUC: 6 of 20 miRNAs and ANOVA: 6 of 19 miRNAs) being present in greater than 50% of the models and did not change when the validation set AUC was required to be greater than 0.8. Cox proportional hazards (CoxPH) models were built using the expression of each 6-miRNA set across all 48 samples. **C**, **D**: The predictive ability of each 6-miRNA set in B was assessed by ROC curve and AUC based on calculated risk score. Hsa-miR-7855-5p was computationally excluded from the final ANOVA miRNA set model. 95% confidence intervals (CI) are indicated by gray area around each curve. Sensitivity is the true positive rate and 1-specificity is the false positive rate. **E**-**H**: Graphical representation of risk scores for each of the 48 samples based on a CoxPH model generated from the 6-miRNA AUC set (E, G) or 5-miRNA ANOVA set (**F, H**). Dotted line is the threshold used to distinguish cases from controls. Squares represent eventual breast cancer cases and circles represent cancer-free controls (G, H). p < 0.0001 between cases and controls.

The identified 25 candidate miRNAs (Figure 2D, 2E) were used to develop a risk score. A bidirectional stepwise regression Cox proportional hazards (CoxPH) model was utilized to identify those miRNAs that, when combined, best distinguished cases from controls. We utilized computational methods to account for the limited size of our patient database. Specifically, the 48 patients were randomly divided into a training set of 32 samples and a validation set of 16 samples and 1000 individual models generated (Figure 3A). For each miRNA set (AUC or ANOVA sets), a CoxPH model was built using only the expression levels of miRNAs from each set in the randomly selected patient training set (n=32). The model was then tested on the remaining patient validation set (n=16) and evaluated by the AUC. This process was repeated for 1000 iterations (Figure 3A). It is important to note that nearly 70 billion potential combinations exist in selecting training and validation sets. Candidate signature miRNAs were model-selected based on AIC and refined by 2 criteria: 1) presence in over 500 models and; 2) presence in over 50% of the models with an AUC > 0.8 in the validation patient set. Nine out of 25 miRNAs passed these thresholds with each miRNA set containing 6 miRNAs (Figure 3B). Three miRNAs (hsa-miR-1184, hsa-miR-4423-3p, and hsa-miR-7855-5p) were common to both the AUC and ANOVA sets.

Final models were generated separately for the top model-selected miRNAs, 6 for AUC set and 6 for ANOVA set (Figure 3B), utilizing expression levels across all 48 patients. Each set of 6 miRNAs in Figure 3B was input into our algorithm and ROC curves generated. While all 6 miRNAs in the AUC set were used to generate a risk score, hsa-miR-7855-5p was computationally excluded in the ANOVA set, based on AIC model selection, as the addition of this miRNA did not improve the model in ability to classify patient outcome. The miRNA-modeled risk scores performed well at classifying cases, with AUC and 95% confidence intervals of 0.896 (0.804-0.988) and 0.870 (0.771-0.970) (Figure 3C, 3D). The models generated the following risk score formulas:

**Formula 1: AUC-selected miRNA-modeled risk score**

Risk score = (-1.062 x hsa-miR-3124-5p) + (-0.32 x hsa-miR-1184) + (-0.33 x hsa-miR-4423-3p) + (0.621 x hsa-miR-4529-3p) + (-0.626 x hsa-miR-7855-5p) + (0.359 x hsa-miR-4446-3p)

**Formula 2: ANOVA-selected miRNA-modeled risk score**

Risk score = (-0.274 x hsa-miR-1184) + (-1.305 x hsa-miR-766-3p) + (-0.393 x hsa-miR-4423-3p) + (0.601 x hsa-miR-4727-3p) + (0.229 x hsa-miR-208a-5p)

These formula-generated risk scores were applied to miRNA levels from each case and control for discriminatory power. Figure 3E through H demonstrate that the risk scores clearly distinguishes cases from controls as illustrated by the model-calculated threshold (Figure 3E-3H, dotted line). This threshold can be used to identify women who are at significant risk for developing breast cancer. Associated model statistics are reported in Table 2.

**Table 2:** Descriptive statistics of each miRNA within iterative models

	Coefficient	p-value	# of models (n = 1000)	# of patients detected in (n = 48)
**Modeled miRNAs from Top 20 AUC miRNAs**				
hsa-miR-3124-5p	-1.062	5.3E-05	817	23
hsa-miR-1184	-0.32	0.01044	557	47
hsa-miR-4423-3p	-0.33	0.00946	619	44
hsa-miR-4529-3p	0.621	0.00029	674	39
hsa-miR-7855-5p	-0.626	2.4E-05	663	41
hsa-miR-4446-3p	0.359	0.01243	622	17
**6-miRNA signature AUC (95% CI) = 0.896 (0.804 – 0.988)**				
**Modeled miRNAs from 19 ANOVA p < 0.05 miRNAs**				
hsa-miR-1184	-0.274	0.00998	575	47
hsa-miR-766-3p	-1.305	0.00021	779	11
hsa-miR-4423-3p	-0.393	0.00174	793	44
hsa-miR-4727-3p	0.601	0.02527	672	8
hsa-miR-208a-5p	0.229	0.07624	617	4
**5-miRNA signature AUC (95% CI) = 0.870 (0.771 – 0.969)**				

**Figure 4:**
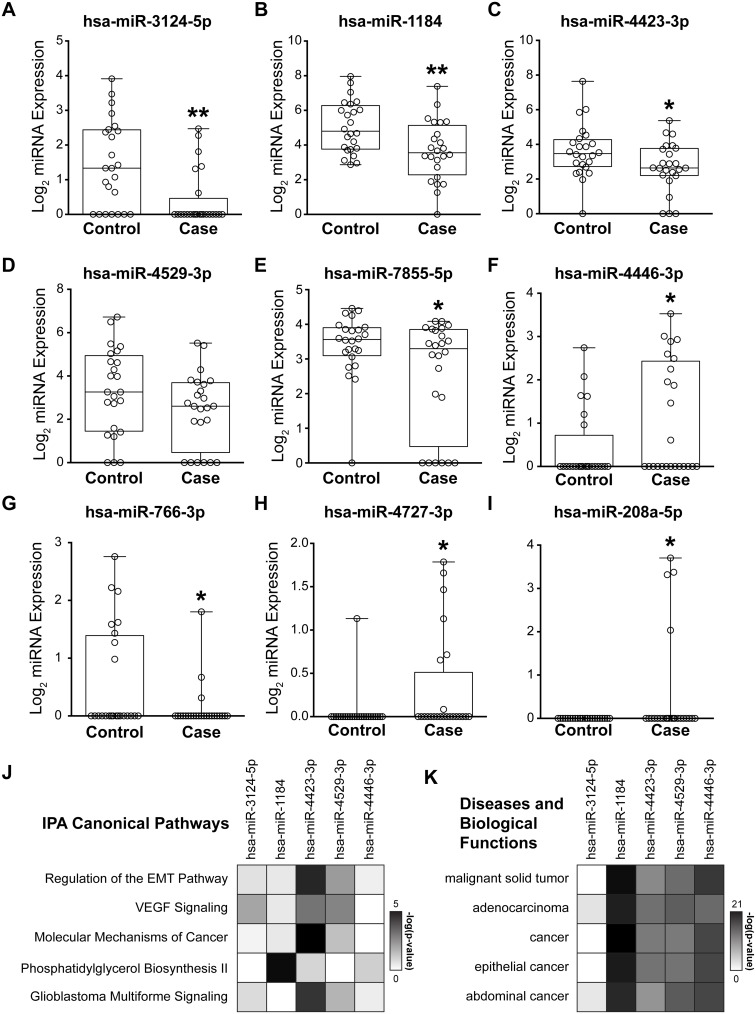
Biological implications of risk signature miRNAs **A**-**I**: Box and whisker plots of normalized log_2_ microarray expression for 9 candidate miRNAs identified through an iterative multivariable CoxPH model. Each open circle represents an individual patient. A-F: 6 AUC set miRNAs ordered from high to low individual AUC. B, C, G-I: 5 ANOVA set miRNAs ordered from low to high p-value. G-I: The 3 miRNAs unique to the ANOVA set are only detected in a small percentage of samples and were not included in downstream pathway analysis. **J**, **K**: Targets of the 6 AUC set miRNAs were identified in Ingenuity Pathway Analysis (IPA, www.ingenuity.com) and core analyses run. No genes were present in the interaction network for hsa-miR-7855-5p. The top 5 enriched IPA canonical pathways (J) and diseases and biological functions (K) were identified through comparison analysis of these miRNA interaction networks. Colors in the heat maps are based on the -log(p-value) of enriched pathway over genomic background where a value > 1.3 corresponds to p < 0.05. n = 24 controls and n = 24 cases. * p < 0.05 ** p < 0.01 for both paired (matched case/control) and unpaired t-tests.

We further evaluated the expression of all model-identified miRNAs used for risk score calculation, a combined 9 individual miRNAs, in all 48 patient samples (Figure 4A-4I). Two-thirds of these miRNAs, hsa-miR-3124-5p (Figure 4A), hsa-miR-1184 (Figure 4B), hsa-miR-4423-3p (Figure 4C), hsa-miR-4529-3p (Figure 4D), hsa-miR-7855 (Figure 4E), and hsa-miR-766-3p (Figure 4G), tended to have lower expression levels in cases, compared with controls. Conversely, hsa-miR-4446-3p (Figure 4F), hsa-miR-4727-3p (Figure 4H), and hsa-miR-208a-5p (Figure 4I) were detected at elevated levels in cases, compared with controls. Of note, the 3 miRNAs unique to the ANOVA set (hsa-miR-766-3p, hsa-miR-4727-3p, and hsa-miR-208a-5p) were detected in less than 25% of patients (Table 2, Figure 4G-4I). Thus, we eliminated these miRNAs from our final risk signature, as they are not ideal risk markers to transition to the clinic.

### Biological Pathway Analysis

Biological pathway analysis was performed for genes targeted by miRNAs identified in the risk signature to elucidate potential mechanistic roles in breast cancer development. We focused our analyses on the 6 miRNAs frequently detected in serum of at-risk women that were also identified from the AUC-selected miRNA set (Figure 3B, 3C, 3E, 3G and Figure 4A-4F). The interaction networks of hsa-miR-3124-5p, hsa-miR-1184, hsa-miR-4423-3p, hsa-miR-4529-3p, hsa-miR-7855-5p, and hsa-miR-4446-3p were identified via Ingenuity Pathway Analysis (IPA, www.ingenuity.com). No targets exist within IPA for hsa-miR-7855-5p. Thus, it was excluded from pathway analysis. We used the comparison analysis feature in IPA to discover biological pathways regulated by the remaining 5 miRNAs in combination. Regulation of the Epithelial-Mesenchymal Transition Pathway, VEGF Signaling, and Molecular Mechanisms of Cancer were within the top 5 Ingenuity Canonical Pathways indicating enrichment in genes targeted by risk signature miRNA in cancer-related biological processes. (Figure 4J). Further, all of the top 5 enriched Diseases and Biological Functions are directly tied to cancers (Figure 4K). The difference in these risk-associated miRNAs and predicted deregulated pathways may predispose women to develop breast cancer, providing both novel biomarkers as well as insight into avenues for breast cancer-prevention.

## DISCUSSION

Using samples obtained from women years prior to being diagnosed with breast cancer and an iterative strategy for modeling, we have discovered a 6-miRNA signature of breast cancer risk. This 6-miRNA signature distinguishes cases from controls in a cohort of clinically similar high-risk women. Additionally, the miRNAs taken together were more informative then any single miRNA. Some of the miRNAs in this signature are involved in many cancer-related pathways. To our knowledge this is the first signature of breast cancer risk using circulating miRNAs and may represent an important “liquid biopsy” for identification of women at greatest risk for developing breast cancer. Additionally, the functions of the miRNAs in our signature may identify novel targets for prevention strategies.

The 6-miRNA signature of miR-3124-5p, miR-1184, miR-4423-3p, miR-4529-3p, miR-7855-5p, and miR-4446-3p performs better than current models with an AUC of 0.896 (CI 0.804-0.988) (see Figure 3). In published studies the Gail model has been found to have modest performance with an AUC of 0.55-0.62 [29–31], while the Claus model appears to perform somewhat better with an AUC of 0.71 [5]. The IBIS model is thought to be a more generalizable model, given that family history, biopsy history and other factors are included. Published studies demonstrate AUCs between 0.54-0.76 for this model [5, 6]. A number of efforts have been made to improve the current models or develop new models with individualized factors such as breast density or single nucleotide polymorphisms (SNPs). These newer or refined models have shown small improvement, but accuracy remains (AUC < 0.75) [32–35]. Given the high performance of this 6-miRNA signature, it would appear to have significant clinical applicability for risk prediction.

The classification ability of each miRNA in the signature was lower (AUC ranging from 0.632 to 0.766, Supplementary Table 1) than that of the 6-miRNA signature together (AUC=0.896). The majority of published studies investigating use of miRNAs in breast cancer have focused on the discriminatory value of single miRNAs [24]. However, recent studies have recognized that a signature of several markers such as miRNAs will be less vulnerable to biological differences and therefore more valuable for clinical use. The use of miRNA signatures for cancer risk prediction has shown promise for colon, lung and prostate cancer [36–38].

To our knowledge, our findings represent the first miRNA signature associated with breast cancer risk, although several studies have identified miRNAs associated with early detection of breast cancer [25–28]. Taslim et al. identified a group of miRNAs from breast tissue and validated the predictive value of a 20-miRNA signature in serum obtained <18 months prior to diagnosis [27]. Godfrey et al. also identified a different set of miRNAs associated with a breast cancer diagnosis in women <18 months prior to diagnosis [25]. Interestingly, the two studies derived their case-cohort study from the same population and it would appear they utilized the same cases and controls but identified different miRNAs associated with risk, which may suggest methodological issues. Chang and colleagues identified 5 miRNAs differentially expressed between cases and controls a mean of 1 year prior to diagnosis [28]. However, in the validation set (with a mean time from serum collection to diagnosis of 78 months) significance was not achieved; suggesting these miRNAs may be more appropriate for early detection. Muti and colleagues identified 2 miRNAs that may be associated with longer term risk among postmenopausal women [26]. Both Chang and Muti used whole blood, which is not recommended for biomarker analysis given that miRNAs may be released from multiple cell types within whole blood [24].

Among the 6 signature miRNAs detected in more than 25% of high-risk women in the present study, only miR-4446-3p is upregulated while the other 5 are downregulated in cases as compared to controls. The miRNA with the highest individual AUC in our signature, miR-3124-5p, has been associated with triple negative breast cancer [39], as well as other cancers [40, 41]. Published studies demonstrate that miR-1184 (expressed at the highest levels in our high-risk cohort), is located on the X chromosome but has not been studied in breast cancer. Circulating levels of miR-1184 are reported to be increased in patients with prostate cancer [42], but were decreased in breast cancer cases in our cohort. MicroRNA-4423-3p has a role in regulating epithelial cell differentiation [43], is reduced in lung tumors [43], and downregulated in rheumatic heart disease [44]. At present, no records for miR-4529-3p exist in PubMed, with our study providing novel cancer association. No functional studies of miR-7855-5p have been reported. However, this miRNA resides in an intron of spectrin; a gene associated with platelets and inflammation [45]. The upregulation of miR-4446-3p in both breast cancer cells and cancer-associated fibroblasts results in loss of tumor suppressor gene expression [46]. Of the three miRNAs present in less than 25% of high-risk women, miR-766-3p is down-regulated, while miR-4727-3p, and miR-208-5p are highly up-regulated. While these 3 miRNAs may function in cancer initiation, they are not ideal biomarkers for clinical screening due to limited detection and low expression values. Future studies will characterize the role of the identified signature miRNAs on promoting breast cancer development.

### Strengths

While we acknowledge that our miRNA signature must be tested in a larger, independent cohort, our study has several strengths. We have used archived serum from women who developed cancer a median of 3.2 years after serum was obtained (66.7% (16/24 cases) developed cancer more than 2 years after serum collection). The median follow-up of women in our database is 8.7 years and the control group has been followed for longer (a mean of 11.2 years), suggesting that these controls are truly cancer-free. For this analysis, we used the most comprehensive miRNA microarray available (miRBase v20 – 2578 human miRNAs). Prior studies regarding early detection and risk have largely screened miRNAs known to be associated with breast cancer [20] and/or identified in breast tissue [27]. Use of a more comprehensive miRNA panel provides an unbiased approach, allowing us to identify previously uncharacterized miRNAs and miRNAs more likely associated with breast cancer risk, potentially representing an interaction between host and tumor factors. In fact, there is little overlap of the miRNAs in the 6-miRNA signature with other miRNAs associated with early detection, prognosis or treatment [47]. It is possible that what drives breast cancer risk may not be the same as what drives cancer development. Given that all cells have the potential to be malignant, the development and propagation of cancer needs to take into account host and stromal factors as well as other cells responding to tumor initiation [48].

### Limitations

There are limitations of our study which must be acknowledged. Our study is small and validation in a larger, more diverse dataset is critical. We have secured access to samples from 3 large randomized chemoprevention trials (MAP.3, NSABP-P1 and NSABP-P2) and will be testing our findings in the placebo arms of these trials. The AUC that we have identified is quite high and we anticipate that testing in a validation cohort will result in some loss of accuracy. Elimination of the 3 miRNAs that were poorly expressed (likely due to the small size of our sample) is a potential limitation of our model and this will be addressed in future validation studies by using a larger number of cases and controls and screening for all 25 candidate miRNAs (see Figures 2D-2E and 4G-4I). The use of a 2578 miRNA panel (Affymetrix GeneChip miRNA v4) as opposed to a panel of miRNAs chosen for association with breast cancer could be considered a limitation as this represents a non-targeted approach. However, miRNAs previously identified with association in breast cancer (i.e., lower numbered miRNAs) are included in the Affymetrix v4 panel. Additionally, as outlined above, the use of this panel represents a less biased approach allowing us to identify a novel miRNA signature which incorporates yet to be described factors associated with breast cancer risk.

## MATERIALS AND METHODS

### Patient and sample identification

The High-Risk Breast Program (HRBP) at the University of Vermont Cancer Center is a prospective cohort of women at increased risk for developing breast cancer due to one or more of the following risk factors: a strong family history, benign breast disease, prior irradiation for Hodgkin's disease, a known pathogenic mutation in a breast cancer-causing gene, and/or a modeled lifetime breast cancer risk of over 20% at time of enrollment. All participants are recruited from the high-risk breast clinic where they receive screening recommendations according to individual risk and clinical guidelines. Enrollees provide written informed consent to be included in the HRBP database and re-contacted every 4 years thereafter for follow-up. At baseline and subsequent follow-up visits, blood samples are obtained and data collected via questionnaires and medical records to update reproductive and family histories, breast imaging results, lifestyle and health behaviors. Medical records and pathology reports are reviewed at each visit to ascertain incident breast cancers in the cohort. Serum is obtained from coagulated whole blood samples by centrifugation at 1811 g for 10 minutes. Serum aliquots are stored at -80°C within 1 hour of blood draw. Since 2003, 605 women have been enrolled in the HRBP and followed for a median of 8.7 years (range 0.06 – 13.7 years) and enrollment is ongoing. To date, forty-four women have been diagnosed with breast cancer, for an age-adjusted invasive breast cancer incidence of 367/100,000 women years (compared to the age-adjusted rate in the US of 125/100,000).

For the current study, cases and controls were identified from the above cohort. All HRBP participants who were diagnosed with invasive breast cancer more than 6 months after enrollment were selected as cases, with the following exceptions: we excluded women who developed either non-invasive (Stage 0) or metastatic (Stage IV) breast cancer, had a pathogenic mutation in a cancer-causing gene, were diagnosed with another cancer prior to their diagnosis of breast cancer, or for whom serum was not available. Controls were randomly selected from HRBP participants who were cancer free and matched on both age at serum sampling (+/- 3.5 years) and qualifying risk factor for HRBP enrollment (i.e. strong family history or benign breast disease).

### Abstracted clinical information

Data were abstracted from subjects’ electronic medical records to complete information needed for risk model calculation (i.e., reproductive factors), to calculate the Charlson Comorbidity Index [49], and to update time-varying factors (e.g. genetic testing results). Breast density was abstracted from the mammogram report closest to the blood draw (+/- 4 years).

### RNA isolation

Total RNA was isolated from patient serum using a standardized protocol [50]. Serum from 3-6 women were processed in batches. RNA was isolated from a 200μL serum aliquot using the miRNeasy Serum Plasma kit with ce-miR-39 spike-in (QIAGEN), QIAcube (QIAGEN) automation, and eluted with 14μL of nuclease-free water. Multiple serum aliquots from the same patient were processed simultaneously, RNA pooled, and stored in small aliquots to isolate sufficient RNA for downstream applications.

### Global circulating miRNA screen

Exactly 8μL of isolated RNA was prepared for Affymetrix GeneChip miRNA v4 microarrays (Thermo Fisher Scientific) following manufacturer's recommended protocol except for an increased hybridization time of 42 hours to increase signal-to-noise ratios. The data have been deposited into GEO datasets (GSE98181).

### Microarray probeset summarization and normalization

Raw *.CEL data files were processed individually using the apt-probeset-summarize function within the Affymetrix Power Tools v1.18.0 (APT) software package (Thermo Fisher Scientific). Simplified Expression Analysis (SEA) algorithm was used to summarize probesets and the Detectable Above BackGround (DABG) algorithm was used to assign p-values to probeset intensities. All annotation and library files for the miRNA v4 GeneChips were downloaded via the NetAffx Analysis Center (www.netaffx.com - Thermo Fisher Scientific). The command line code used is: apt-probeset-summarize -c miRNA-4_0.clf -p miRNA-4_0-st-v1.pgf -b miRNA-4_0-st-v1.bgp --qc-probesets miRNA-4_0-st-v1.qcc -a pm-gcbg,sea -a pm-gcbg,dabg

Following probeset summarization, the average of 95 anti-genomic probesets was subtracted from each RNA expression probeset in R v3.x [51]. Any value less than 0, below microarray background, was set to 0. For present/absent calls, all probesets with a DABG p > 0.05 were set to 0. These background normalized values were log_2_ transformed. The R script for this normalization is included as a supplement.

### Receiver operator characteristic (ROC) curves

ROC curves and associated area under the curve (AUC) and 95% confidence interval (CI) values were generated for each tested parameter (clinical characteristic, individual miRNA expression, combined miRNA model score) using the pROC v1.8 R/CRAN package [52]. The complete 48-patient study cohort was used to generate ROC curves.

### Building a Cox proportional hazards model

The normalized log_2_ expression values for all 2578 mature miRNAs as well as clinical classification as case or control for each patient were imported into R. Univariate Cox proportional hazards (CoxPH) models for each miRNA were generated starting with an otherwise blank model and the p-value calculated using the coxph function of the survival v2.39-5 R/CRAN package [53, 54]. Prior to multivariable model generation, data were restricted to: 1) the top 20 miRNAs based on highest individual AUC; 2) 19 miRNAs with expression that differed significantly between cases and controls (ANOVA, p<0.05). The stepAIC function in the MASS 7.3-45 R/CRAN package [55] was used for stepwise regression in both forward and backward directions to identify those miRNAs which, in combination, best distinguished cases from controls. Risk scores (log hazard ratio) were calculated using miRNA expression levels and corresponding regression coefficients from multivariable models.

### 1000 model iterations using randomly selected training and validation datasets

The 48 patient samples were randomly divided into a 1000 different training sets of 32 and validation sets of 16. For each training set, a CoxPH model was built as described, signature miRNAs identified, and risk scores and AUCs calculated. Using these signature miRNAs, risk scores and AUCs were calculated for the validation set.

### Final model generation

MicroRNAs identified in at least 500 models with a validation set AUC > 0.8 in greater than 50% of the models were selected and used to generate a new CoxPH model with associated risk scores. All 48 patient samples were used in final model generation.

### Biological pathway analysis

Interaction networks for each miRNA were identified in Ingenuity Pathway Analysis (IPA, www.ingenuity.com) and core analyses run using default parameters. A comparison analysis combining each individual miRNA target analysis was run. The top 5 Ingenuity Canonical Pathways and Diseases and Biological Functions were identified based on maximum values of the negative logarithms of p-values.

### Statistics, graphical representation, and figure preparation

Statistics and graphing were performed in Prism 6 (GraphPad), R v3.x, or Spotfire v2 (Tibco). Density plots were generated using the sm.density.compare function of the sm v2.2-5.4 R/CRAN package [56]. All R scripts used are included as a supplement. Heatmap values and principal component analyses were generated in Spotfire v2 (Tibco). All data were imported into Adobe Illustrator CC for final figure creation.

## CONCLUSIONS

We have identified a 6-miRNA signature which can discriminate between high-risk women who do and do not develop breast cancer over a median follow-up of 8.7 years. The accuracy of this signature is greater than that of any published risk models. Given the accessibility and stability of circulating miRNAs, they have the potential to significantly improve breast cancer risk prediction by identifying women at greatest risk who can be offered aggressive screening, prevention and clinical trials. Future studies by our group will focus on testing in larger, independent cohorts from completed clinical trials and functional analysis of the miRNAs which make up this signature.

## SUPPLEMENTARY MATERIALS FIGURES AND TABLES








