# Supplementary material for: Development of a predictive miRNA signature for breast cancer risk among high-risk women
Source: Oncotarget. 2017 Nov 28;8(68):112170–83. doi: 10.18632/oncotarget.22750 (PMC5762501; doi:10.18632/oncotarget.22750)
Supplement: Supplementary file 1 [file oncotarget-08-112170-s001.pdf]

Development of a predictive miRNA signature for breast cancer risk among high-risk women

SUPPLEMENTARY MATERIALS

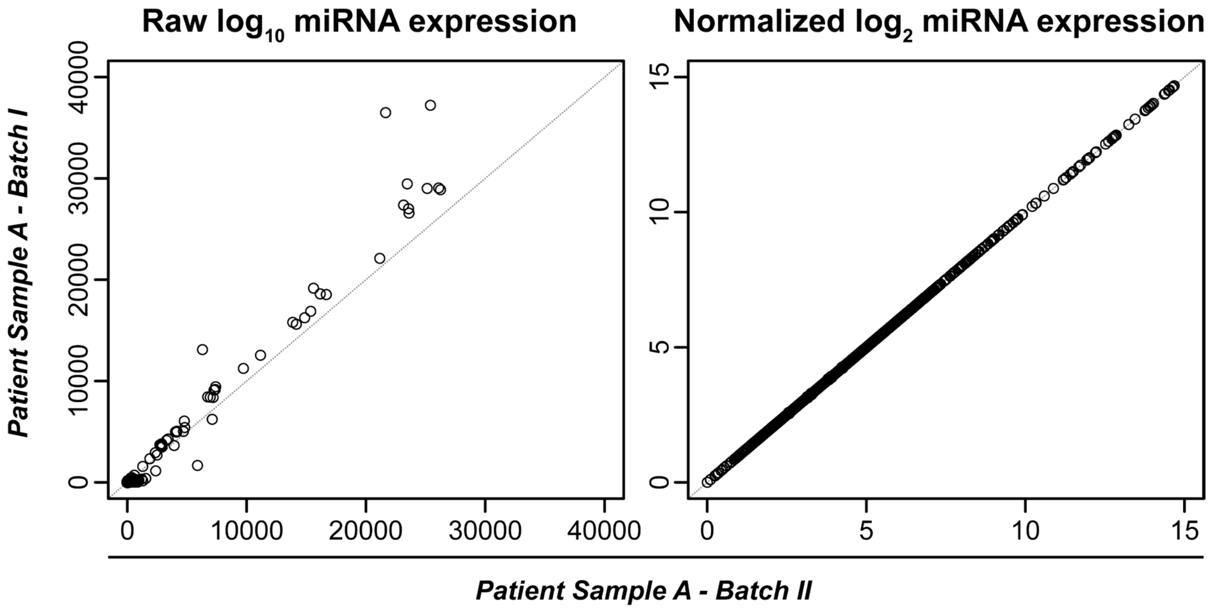

Supplementary Figure 1: Normalization strategy eliminates user and batch effects. Two 1mL serum aliquots collected from the same woman on the same day were processed separately to assess the normalization strategy. Briefly, RNA was isolated from each 1mL aliquot on different days by researchers in separate labs. Subsequently, RNA was processed and hybridized onto Affymetrix microarrays a month apart. Raw miRNA expression values are similar but not identical. Following our normalization strategy, we observe a 1:1 correlation between the 2 samples.

Supplementary Tables see in Supplementary Files.

Supplementary Table 1: The AUC and 95% confidence interval (CI) was calculated for each miRNA to assess the ability of each miRNA to classify cases from controls. MicroRNAs are ordered from largest AUC to smallest AUC.

Supplementary Table 2: An analysis of variance (ANOVA) was performed to test for differences in miRNA expression between cases and controls. MicroRNAs are ordered from lowest to highest p-value.
